# Three-pressure prediction method of jointing well-seismic data in JT1 well area of Sichuan Basin in China

**DOI:** 10.1038/s41598-024-67085-8

**Published:** 2024-07-11

**Authors:** Hu Zhao, Zhong-wei Zhang, Rong-rong Zhao, Wei Chen, Chang-long He

**Affiliations:** 1 Natural Gas Geology Key Laboratory of Sichuan Province, Southwest Petroleum University, Chengdu, 610500 China; 2 School of Geoscisence and Technology, Southwest Petroleum University, Chengdu, 610500 China; 3 Exploration Division, Petrochina Southwest Oil&Gas Field Company, Chengdu, 610041 China

**Keywords:** Sichuan Basin, Maokou formation, Pressure prediction, Formation pressure, Pre-stack inversion, Geology, Geophysics

## Abstract

With the high yield of many wells represented by Well JT1 in the Maokou Formation, has catalyzed a surge in exploration activities along the platform margin facies of the Maokou Formation in central Sichuan and further showed the significant exploration potential of the Maokou Formation in the northern slope. However, the fracture cave body of the Maokou Formation exhibits a high degree of development, strong longitudinal and horizontal heterogeneity, large formation pressure differences, and drilling events such as gas kicks and lost circulation occur frequently, which seriously affects the efficient implementation of drilling. Understanding the spatial distribution of the three-pressure in the formation can help better deal with and solve the above problems. Therefore, in order to help the safe, high-quality and rapid drilling of the Maokou Formation in the study area, and enhance the efficiency of oil and gas development, this paper explores the research on the prediction method of the three-pressure of jointing well-seismic data based on the geomechanical experimental data and the actual drilling data. In the process of prediction of pore pressure, this study found that the pore pressure and formation velocity in the study area have an exponential relationship. In order to enhance the applicability of the Filippone’s method in the study area and improve the prediction accuracy of pore pressure, the linear relationship between pore pressure and formation velocity in the Filippone’s method is modified to an exponential relationship, and a pore pressure prediction model suitable for the work area was established. Based on the Mohr–Coulomb criterion and Huang's model, the prediction models of collapse pressure and fracture pressure applicable to the study area were established, respectively. Then, the elastic parameters were obtained through pre-stack inversion, and the three-pressure bodies were calculated based on the elastic parameter bodies. The results indicate that: (1) The three-pressure prediction method of the jointing well-seismic data in this paper can predict the formation's longitudinal and transverse pressure anomaly zones in advance. (2) The Maokou Formation in the study area is characterized by abnormally high pressure, to balance the pressure of the high-ground formation, high-density drilling fluid is necessary. (3) The prediction results of three-pressure in this paper are highly consistent with the actual drilling engineering events, which verifies the reliability of the three-pressure prediction results presented in this study. The results of the study can provide a basis for decision-making in drilling geological design, such as the determination of drilling fluid density, the evaluation of borehole stability and other engineering problems that require support from three-pressure data.

## Introduction

With the acquisition of high-yield industrial natural gas from Well JT1 in the Maokou Formation, the exploration boom of the Maokou Formation in central Sichuan was opened, and then many wells obtained high-yield natural gas in the Maokou Formation, further indicating the significant exploration potential of the Maokou Formation on the northern slope. However, due to the influence of surface uplift during the sedimentary period, the Maokou Formation is affected by dissolution, the fracture-cave body is developed, the heterogeneity is strong, the formation pressure and the gas reservoir pressure are very different, and the drilling events such as gas kick, lost circulation, and wellbore collapse occur frequently, leading to increased engineering risks during the drilling process. These challenges significantly impact the geological design of early drilling and the efficient execution of drilling operations in later stages. Obviously, the prediction of the three-pressures (pore pressure, collapse pressure, and fracture pressure)of the Maokou Formation before drilling is particularly important.

At present, methods for predicting formation pore pressure can be divided into two categories: one is based on the normal compaction trend line (equivalent depth method^1^, Eaton’s method^2^), the other is the methods that do not require establishing a normal compaction trend line (Fillippone’s method^3^, improved Fillippone’s method^4,5^, and the effective stress method^6^). The prediction of collapse pressure mainly involves methods based on the Mohr–Coulomb criterion, Hoek and Brown criterion, nonlinear Pariseau criterion, and Drucker and Prager criterion, among which the method based on the Mohr–Coulomb criterion is widely used for collapse pressure prediction^7^. The models for predicting fracturing pressure mainly include Hubbert-Willis’s model^8^, Mathews-Kelley’s model^9^, Eaton’s model^10^, Anderson’s model^11^, Stephen’s model^12^, Huang’s model^13^, among which the Huang’s model is more generally applicable to different areas as it considers more influencing factors. Zhu et al. proposed a modification of the Fillippone’s method based on actual measurement data in different work areas to enhance the prediction accuracy^14^. Zhou et al. utilized post-stack impedance inversion to acquire formation layer velocity and achieved abnormal pressure prediction using the Fillippone’s method^15^. Li et al. obtained layer velocity and density through pre-stack inversion, and selected the improved Fillippone’s method for high-precision prediction of pore pressure^16^. Hu et al. established a model for calculating three-pressure in the central block of Dagang oilfield based on Eaton’s method, Mohr–Coulomb criterion and three-dimensional stress model using conventional well logging data and actual measurement data of three-pressure^17^. Su calculated the formation pore pressures of sandstone and carbonate formations by using the Eaton’s method and the effective stress method, respectively, and established a prediction model of fracture pressure and a collapse pressure prediction model considering the effective membrane pressure based on the tensile stress failure theory and the Mohr–Coulomb criterion^18^. Ma et al. used Eaton’s method to predict shale pore pressure and introduced various anisotropic coupling factors based on the Huang’s model to establish a fracturing pressure prediction model suitable for shale formations^19^. Li established a three-pressure prediction model for carbonate rock formations through laboratory acoustic experiments. The model combines effective stress principles, the Mohr–Coulomb criterion, and the maximum tensile pressure failure criterion^20^. Qin et al. respectively predicted the formation pore pressure of continental and marine formations using the equivalent depth method and effective stress method, and eventually calculated the collapse pressure and fracturing pressure based on Mohr–Coulomb criterion and multiple fracturing pressure prediction models^21^. Li took into account the influence of lithology and used Eaton’s method and effective stress method to predict pore pressure of different lithologic formations. Based on laboratory rock physics experimental data, they calculated the construction pressure coefficient using the Huang’s model and eventually obtained collapse pressure and failure pressure based on Mohr–Coulomb criterion and maximum tensile stress failure criterion^22^.

It can be observed that there is relatively little research on the spatial prediction of three-pressure in the strata of the Sichuan Basin, and even less on the prediction of three-pressure in the karst strata of the Maokou Formation. Only Fan et al. (2023) utilized logging data and on-site drilling data to choose the Fillippone’s and Eaton’s method to achieve better prediction of pore pressure across the entire well section of the JT1 well area. However, they only developed a single well pore pressure profile and did not establish a spatial distribution prediction method for the three-pressure in this area^23^. In this paper, based on logging data and indoor rock mechanics experimental data and drilling data, we conducted a joint well-seismic data prediction method for the strata in the JT1 well area, and made modifications to the Fillippone’s method according to the characteristics of the overpressure strata of the Maokou Formation in the JT1 well area. Based on the Mohr–Coulomb criterion and Huang’s model, we established a three-pressure prediction model tailored to this area. By utilizing pre-stack seismic inversion, we successfully predicted the rock elastic parameters and spatial distribution of three-pressure in the Maokou Formation within the JT1 well area. This information serves as a foundation for making informed decisions regarding geological drilling design and optimizing drilling operations in this locality.

## Geological settings

**Figure 1:**
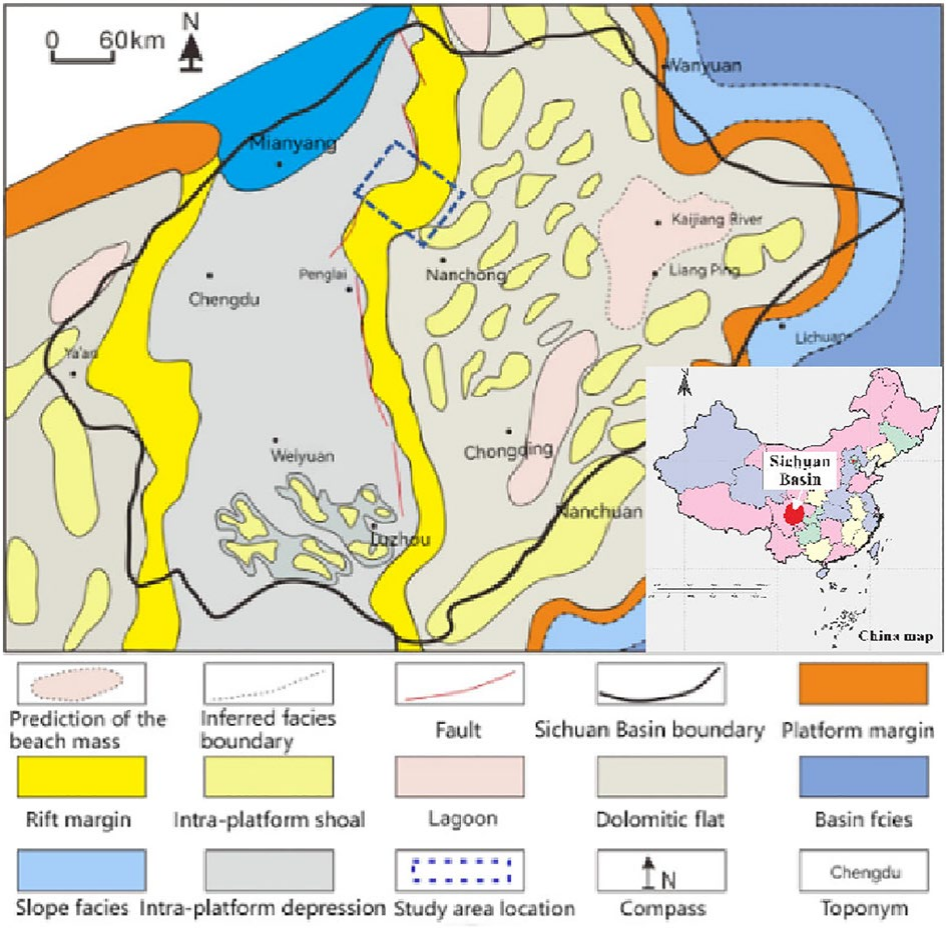
Simplified geologic map of study area.

Due to the effects of dissolution in the Maokou Formation of the JT1 well area, the formation pressure undergoes significant fluctuations, often leading to complications such as mud leakage and wellbore collapse during drilling operations. These issues hinder the economic and efficient execution of drilling activities. Currently, traditional methods predominantly focus on predicting single-well pressure profiles, which cannot accurately achieve pre drilling prediction. In this article, we propose a well seismic joint formation three pressure prediction method, which comprehensively utilizes rock physics testing, well logging interpretation, and pre stack seismic inversion methods for pre drilling prediction of formation three pressure, providing technical guarantees for complex block drilling geological design (Fig. 1).

## Three-pressure prediction principles

### Pore pressure

Pore pressure represents the pressure exerted by the fluid residing within the pores of a formation. It is an indispensable parameter for analyzing the mechanical stability of the wellbore. In combination with the actual conditions of the work area, this article chooses the Fillippone’s method to calculate the pore pressure of the formation. In the 1970s and 1980s, Fillippone proposed an empirical equation for predicting pore pressure using formation velocity, which does not rely on the trend line of normal pressure consolidation. The equation is as follows:

1 $$P_{p} = \frac{{\ln (V_{\max } - V_{i} )}}{{\ln (V_{\max } - V_{\min } )}}*S_{v}$$

where $P_{p}$ is the pore pressure, MPa; $V_{i}$ is the velocity of the prediction interval, m/s; $V_{\max }$ is the velocity of the rock framework, m/s; $V_{\min }$ is the velocity of pore fluid, m/s; $S_{v}$ is the overburden pressure, MPa.

The overburden pressure, a critical parameter in predicting the formation's three-pressure profile, represents the pressure exerted on a geological formation at a specific depth, resulting from the cumulative weight of the overlying rocks and fluids. The following equation outlines the computation of overburden pressure:

2 $$S_{v} = \int_{0}^{H} {\rho \left( h \right)gdh}$$

where $S_{v}$ is the overburden pressure, MPa; $H$ is the burial depth from surface to a specific depth, m; $\rho (h)$ is the density of the overburden rock as a function of burial depth, kg/m^3^; $g$ is the gravitational acceleration, 9.8 m/s^2^.

### Collapse pressure

The pressure that causes a wellbore collapse is called collapse pressure, which is one of the key factors affecting the stability of the wellbore. When the pressure of the fluid column in the well is lower than the formation collapse pressure, the wellbore rocks will undergo shear failure. If the rocks are plastic, they will deform plastically and cause wellbore contraction. If the rocks are brittle, they will collapse and cause wellbore enlargement and stuck pipe. According to the Mohr–Coulomb strength criterion, whether the rocks undergo shear failure is mainly influenced by the maximum and minimum principal pressures that the rocks bear. The specific equation for calculating collapse pressure is as follows:

3 $$B_{p} = \frac{{\eta (3\sigma_{H} - \sigma_{h} ) - 2CK + \alpha P_{p} (K^{2} - 1)}}{{(K^{2} + \eta )}}$$

where $B_{p}$ is the collapse pressure, MPa; $P_{p}$ is the pore pressure, MPa; $\sigma_{H}$ and $\sigma_{h}$ are the horizontal maximum principal stress and horizontal minimum principal pressure, respectively, which are the components of present-day in situ stress in two different horizontal directions, MPa; $C$ is the cohesion of rock, which is the mutual attraction between adjacent parts within the same substance, MPa; $\alpha$ is the biot coefficient,$0 < \alpha < 1$, which is a parameter that describes the sensitivity of changes in pore water pressure in rocks or soils to changes in effective stress;$\eta$ is the nonlinearity correction coefficient of stress, which is a quantity that adjusts the degree of influence of in situ stress on the collapse pressure of the formation; $K = ctg(45^{^\circ } - \varphi /2)$, $\varphi$ is the internal friction angle of rock, which is the angle between the combined force of the normal stress on the ultimate equilibrium shear plane and the internal friction force formed during rock failure and the normal stress.

### Fracture pressure

The formation fracture pressure signifies the maximum tolerable pressure that rocks within a formation can endure when subjected to external forces. Accurately predicting the fracture pressure can effectively prevent drilling accidents such as gas kick, blowout, collapse. Taking into account the geological characteristics of the study area, this paper chooses to calculate the formation fracture pressure using Huang Rongzun's method, which considers the tensile strength. Professor Huang Rongzun advocates that the formation's fracture is determined by the pressure state of the wellbore and takes into account the non-uniformity that exists underground, as well as the influence of the pressure field and the strength of the formation itself. The specific equation is as follows:

4 $$P_{f} = \left( {\frac{2\mu }{{1 - \mu }} + 3\beta_{1} - \beta_{2} } \right)(S_{v} - P_{p} ) + P_{p} + S_{t}$$

where $P_{f}$ is the fracture pressure, MPa; $\mu$ is the Poisson’s ratio;$\beta_{1}$ and $\beta_{2}$ are the coefficients reflecting the horizontal maximum and minimum tectonic stress, respectively, both $\beta_{1}$ and $\beta_{2}$ represent the additional effective stress in the horizontal direction generated by tectonic movements, with different values in different regions; $P_{p}$ is the pore pressure, MPa; $S_{v}$ is the overburden pressure, MPa; $S_{t}$ is the tensile strength of rock, which refers to the maximum stress that the rock can withstand under tensile conditions, MPa.

### Modification of Fillippone method

Fillippone discovered through research that there is a linear relationship between formation pore pressure and velocity. The relationship can be expressed as follows:

5 $$P_{p} = \frac{{V_{\max } - V_{i} }}{{V_{\max } - V_{\min } }}*S_{v}$$

However, the actual formation pore pressure may not necessarily have a linear relationship with formation velocity. Liu et al. found that in the depth range of medium to shallow with relatively small abnormal pressure amplitude, the formation pore pressure and velocity have a logarithmic relationship. Therefore, he modified the Fillippone method as follows:

6 $$P_{p} = \frac{{\ln (V_{\max } /V_{i} )}}{{\ln (V_{\max } /V_{\min } )}}*S_{v}$$

Yet, due to the complexity of the actual reservoir pore pressure, both the Fillippone and the Liu method have issues with inaccuracies in many areas. Therefore, based on the Fillippone relationship and the Liu relationship, with the JT1 well area measured pressure data and logging information as a basis, the Fillippone method is modified to Eq. (1).

**Figure 2:**
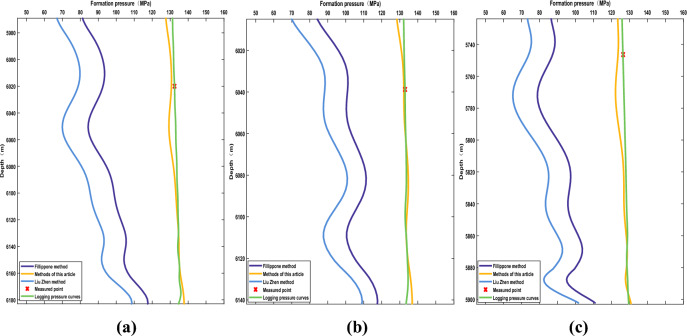
Accuracy analysis of formation pore pressure prediction. (**a**) PY 1 well prediction results. (**b**) PS 7 well prediction results. (**c**) PS 9 well prediction results.

Due to the lack of actual pressure data from well JT1 in the study area, the pore pressure data calculated using laboratory rock physics experiments from wells PS7, PS9, and PY1, as well as the log data calculated pore pressure data, were compared and analyzed to evaluate the accuracy of Eqs. (1), (5), and (6) (Fig. 2 and Table 1). It is evident that the modified Eq. (1) yields the smallest error (yellow line in Fig. 2). Therefore, Eq. (1) is selected as the predictive model for pore pressure in the Maokou Formation in the study area.

**Table 1:** Statistics of the relative error of the prediction results of formation pore pressure in the Maokou formation.

Well name	Depth (m)	Formation pore pressure (MPa)								
		Petrophysical experimental calculations	Predicted value by Filiippone method	Relative error (%)	Predicted value by Liu zhen method	Relative error (%)	The method prediction value of this paper	Relative error (%)	Logging pressure curve	Relative error (%)
PS7	6038.76	132.94	101.04	24.00	88.36	33.53	132.21	0.55	132.88	0.05
PS9	5746.39	126.45	87.23	31.02	73.93	41.53	123.61	2.25	126.35	0.08
PY1	6019.98	132.45	92.56	30.12	78.77	40.53	130.70	1.32	132.44	0.01

Pore pressure is an important component of fracture pressure and collapse pressure, and accurate prediction of pore pressure is the basis for accurately calculating fracture pressure and collapse pressure. Therefore, based on the measured pressure data in the study area, this article establishes a more suitable pore pressure prediction model for the study area, which improves the prediction accuracy of pore pressure, thereby improving the prediction accuracy of fracture pressure and collapse pressure.

### Three-pressure prediction of jointing well-seismic data

**Figure 3:**
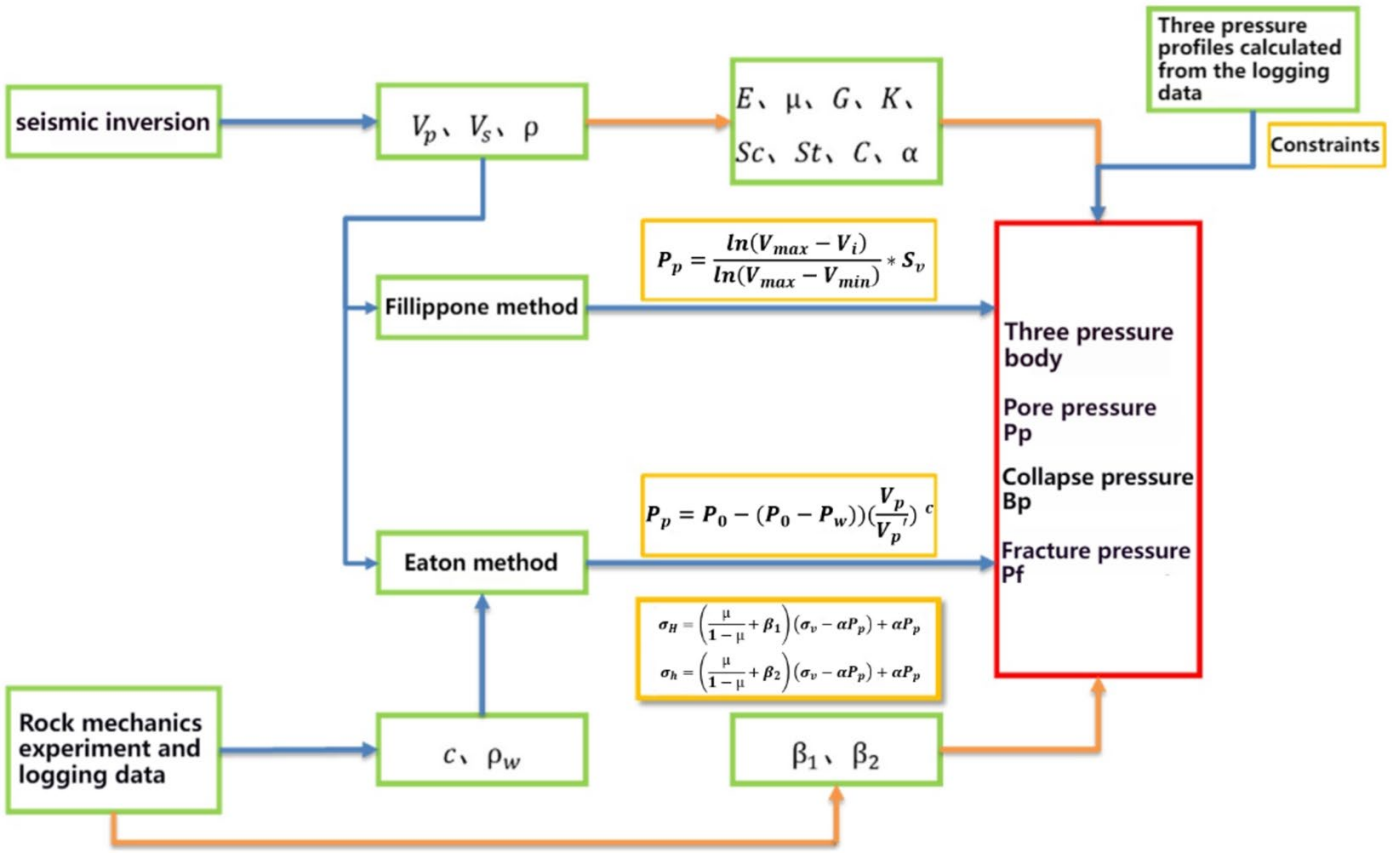
Three pressure prediction flowchart of jointing well-seismic data.

The procedure for integrating well-seismic data for prediction is outlined as follows (Fig. 3): initially, the pressure curve of an individual well is computed using logging data. Subsequently, pre-stack inversion technology is employed to calculate key parameters like P-wave velocity, S-wave velocity, and density. Finally, elastic parameters such as bulk modulus, shear modulus, and Poisson's ratio are calculated based on theoretical equations. Based on the elastic parameters, calculate the three-pressure according to the method outlined in this paper. Subsequently, calibrate and constrain the calculations based on the logging pressure curve and rock physics test results. Calculate the prediction error for each well and utilize this error for fitting to complete the calibration of the three-pressure prediction data.

## Three-pressure prediction of jointing well-seismic data

### Petrophysical characterization

**Figure 4:**
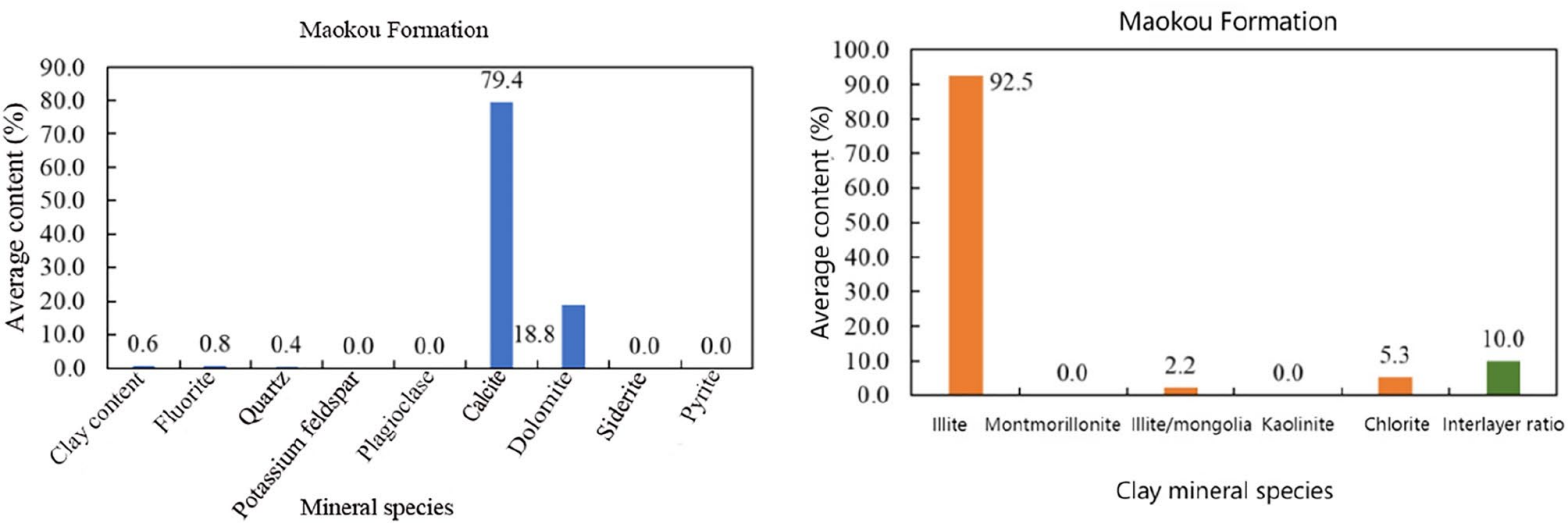
Lithologic characteristics of Maokou Formation.

The results of the rock testing indicate that the lithology of the Maokou Formation in the study area is relatively homogeneous, with the main rock type being carbonate rock and the main mineral being calcite, accounting for 79.4%. The second most abundant mineral is dolomite, accounting for 18.8%, and the average proportion of clay minerals is 0.6%. Among the clay minerals, the primary mineral is illite, accounting for 92.5%, with underdeveloped montmorillonite and kaolinite, and an interlayer ratio of 10%. It is known from the mineral composition of the rocks of the Maokou Formation, carbonate rock of the Maokou Formation is mainly limestone, and the limestone reservoirs have undergone significant changes due to various diagenesis, and the secondary pores often become the main effective reservoir space, thus forming the characteristics of heterogeneity, showing obvious heterogeneity and anisotropy. As an effective reservoir of limestone, the developed fracture system has strong sealing property and is prone to abnormal high pressure. In the process of drilling, if the limestone reservoir develops fractures, it is prone to complex drilling conditions such as blowout and gas kick, and blowout occurs when the drilling fluid density is slightly high, and gas kick occurs when it is slightly lower. Therefore, how to accurately predict the three-pressure of limestone is crucial for the safe drilling of limestone formations (Fig. 4).

**Figure 5:**
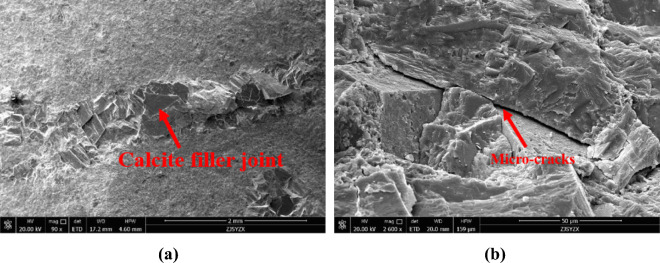
Microstructure characteristics of the Maokou Formation. (**a**) Microscopic pore fracture characteristics of well PY1. (**b**) Microscopic pore and fracture characteristics of Well PY3.

The thin section displays of wells PY1 and PY3 shows that the Maokou Formation is developed with calcite-filled fractures, with a fracture width of about 1 mm. Microscopically, there are microcracks and dissolution pores, and the cracks mainly develop along the cleavage planes of calcite, forming a branching and intertwining pattern. The diameter of the micro-dissolution pores is about 5–20 μm, with smaller pores occurring within grains but densely distributed, while larger intergranular dissolution pores are more susceptible to communication with cracks. Rock is more prone to fracturing at lithological and structural changes. The communication of these pore-throat channels forms interconnected fracture-cavity bodies, creating local pressure anomalies, which may lead to blowout or gas kick accidents. Therefore, it is helpful to conduct pressure prediction and mud density design in advance to prevent drilling accidents (Fig. 5).

The results of rock physics tests in the study area indicate that the average porosity pressure of the Maokou Formation is 133.5 MPa, the average collapse pressure is 126.19 MPa, and the average fracture pressure is 165.40 MPa. The average porosity pressure gradient is 2.224 MPa/100 m, the average collapse pressure gradient is 2.106 MPa/100 m, and the average fracture pressure gradient is 2.756 MPa/100 m.

### Pre-stack elasticity parameter inversion

**Figure 6:**
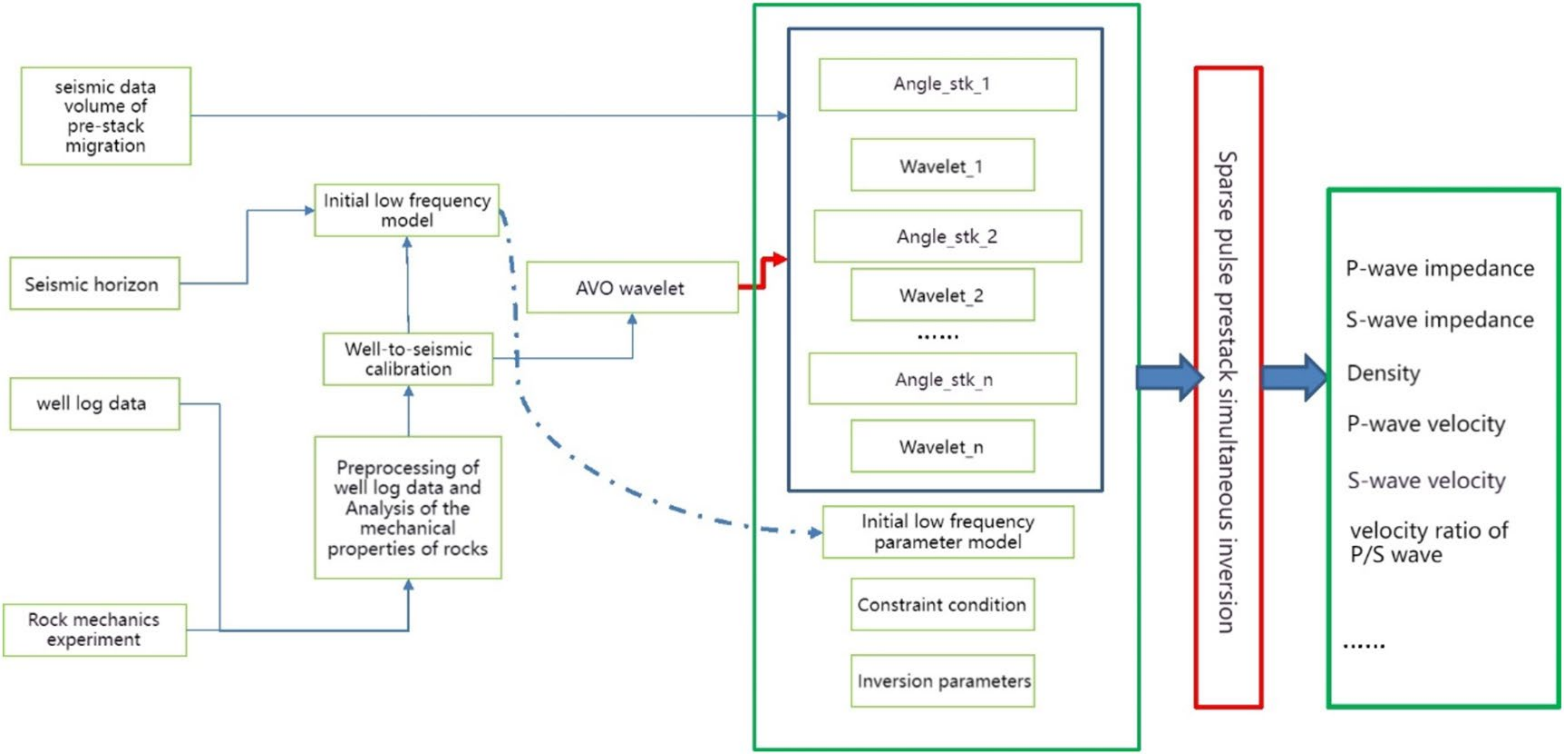
Workflow for AVO constrained sparse pulse pre-stack simultaneous inversion.

**Figure 7:**
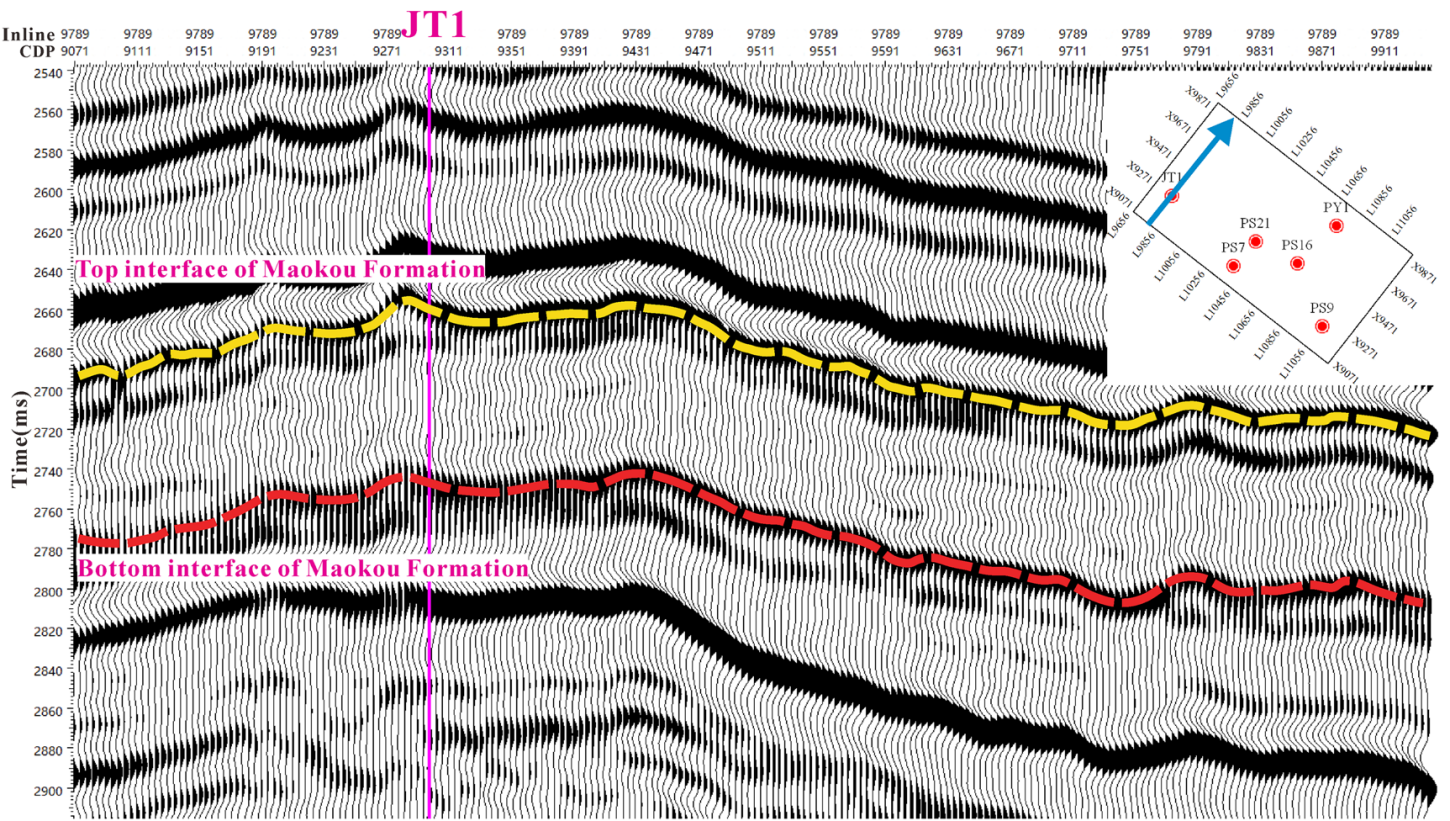
The pre-stack seismic data along with Well JT1.

The pre-stack simultaneous inversion technique, based on AVO-constrained sparse pulse inversion, can derive not only longitudinal wave impedance but also shear wave impedance, density, and a range of other elastic parameters. These parameters are essential for conducting three-pressure prediction. Therefore, in this paper, the AVO-constrained sparse pulse pre-stack simultaneous inversion is used to invert the elastic parameters of the Maokou Formation. The workflow for AVO-constrained sparse pulse pre-stack simultaneous inversion is shown in Fig. 6. Figure 7 displays the profile of the pre-stack seismic data passing through Well JT1 in the study area.

**Figure 8:**
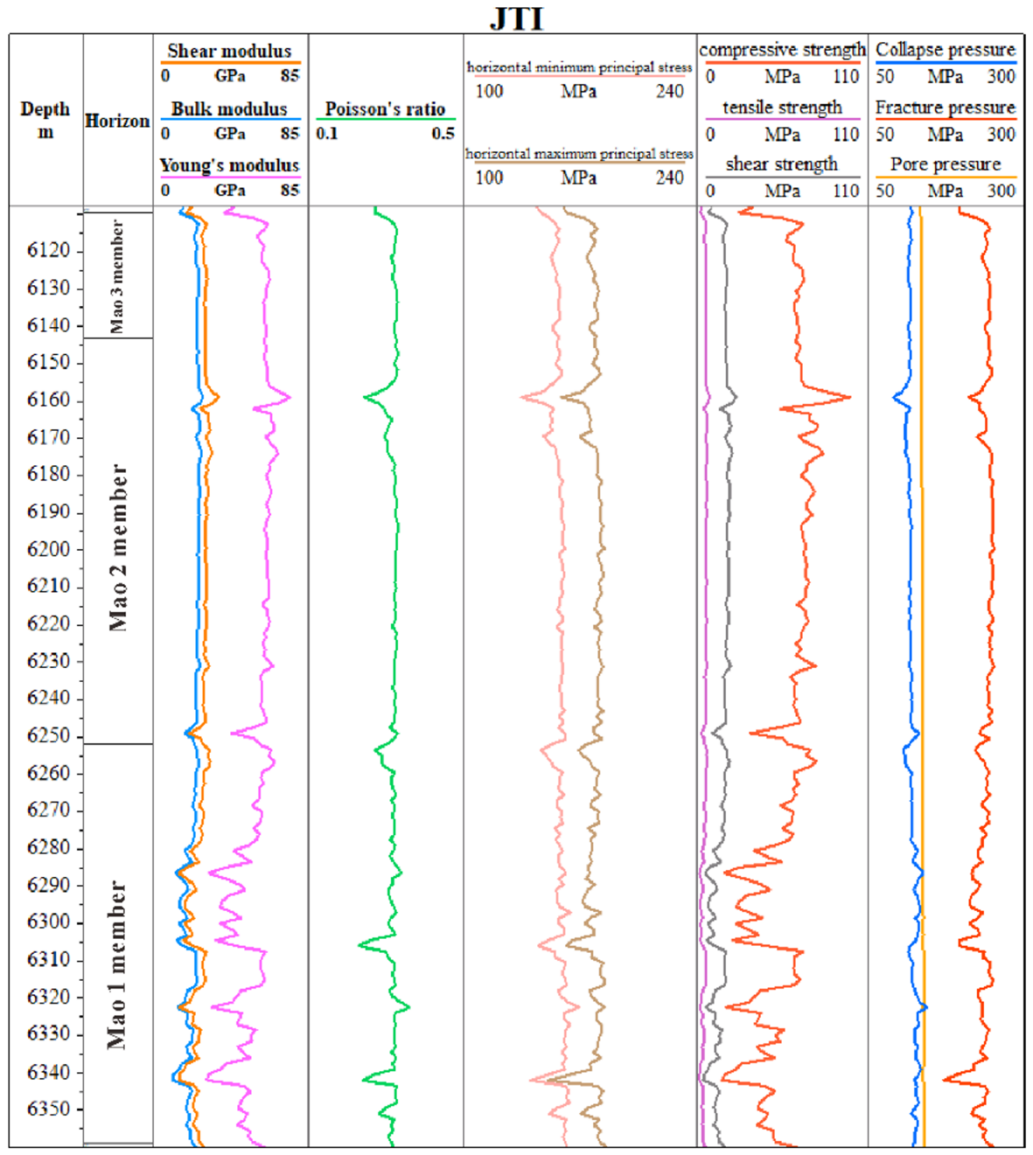
Elastic parameters, pore pressure, collapse pressure and fracture pressure of the Maokou Formation in well JT1 based on well log calculations.

**Figure 9:**
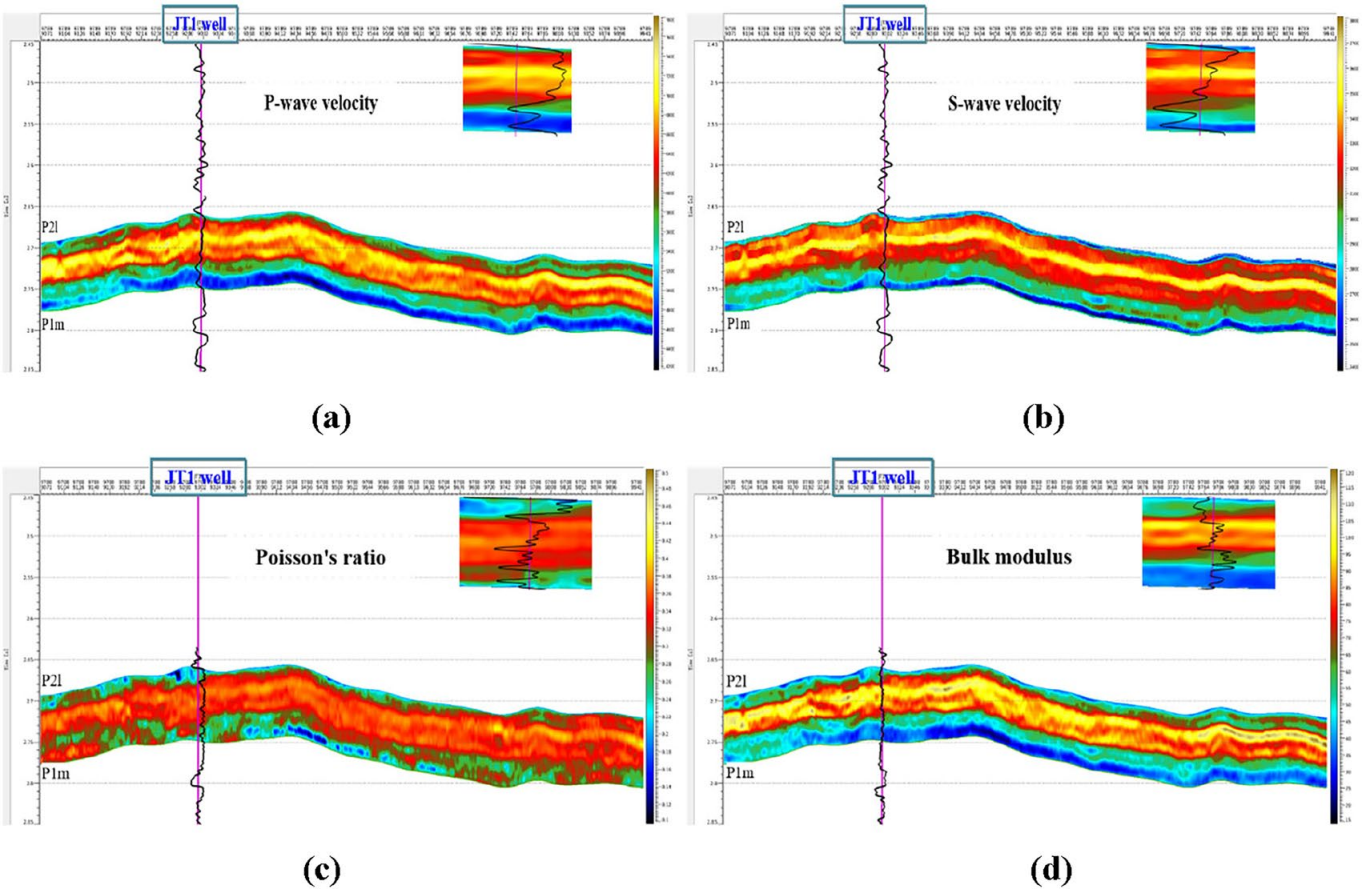
Cross sections of the prestack inversion results of elastic parameters of the Maokou Formation in Well JT1. (**a**) P-wave velocity. (**b**) S-wave velocity. (**c**) Poisson's ratio. (**d**) Bulk modulus.

Using the above prediction method, combined with the pressure curves of 6 wells in the area (Fig. 8), the elastic parameters of the study area can be predicted (Fig. 9). Taking well JT1 as an example, the main elastic parameter statistical table calculated is shown in Table 2.

**Table 2:** Statistical table of pre-stack inversion results of stratigraphic elastic parameters in Maokou Formation.

Horizon	Well name	Depth (m)	Poisson ratio	Young's modulus (GPa)	Bulk modulus (GPa)	Shear modulus (GPa)	Compressive strength (MPa)	Tensile strength (MPa)
Maokou formation	JT1	6109.6–6358.8	0.24–0.37	48.95–94.94	34.81–93.56	19.22–38.01	128.87–249.87	10.74–20.83

To verify the correctness of the calculation results, select some key elastic parameters for verification. First, the dynamically obtained elastic parameters are converted to static parameters through the dynamic-static elastic parameter relationship equation and compared with the rock physics test results. Table 3 shows that the Poisson’s ratio and Young’s modulus parameters calculated by pre-stack inversion are compared with the actual measurements, and the relative error is less than 14%.

**Table 3:** Statistics of the relative error of the inversion results of elastic parameters in the Maokou Formation of Well PS7.

Horizon	Well name	Depth (m)	Petrophysical experiments (average)		Calculation results (average)		Relative error	
			Poisson ratio	Young's modulus (GPa)	Poisson ratio	Young's modulus (GPa)	Poisson ratio	Young's modulus (%)
Maokou formation	PS7	5922.7–6158.1	0.24	50.55	0.26	57.17	8.33	13.10

### Predicted results of three-pressure

The elastic parameters of the Maokou Formation were obtained by pre-stack inversion, and the three-pressure prediction formula established in this paper was applied to finally obtain the three-pressure prediction data, and the three-pressure prediction data body was calibrated and constrained according to the measured data calculated by petrophysical experiments and the three-pressure data calculated based on the logging data of the Maokou Formation, and the accuracy of the three-pressure calculation results was verified by the measured data. Finally, the reliability of the three pressure prediction results is further verified by the coupling analysis of the drilling event and the three-pressure prediction results.

In order to verify the accuracy of different pore pressure prediction methods, rock samples of different depths of three wells were selected for error analysis, Table 4 presents the error analysis table of seismic prediction and logging interpretation and petrophysical test results of Maokou Formation, it can be seen that the modified Philippine method is better than the Eaton method in this study area, and the relative error of the seismic three pressure prediction method in this paper is less than 10%, which verifies that the modified Fillippone method is more suitable for this study area. It can more accurately reflect the local variation characteristics of pore pressure, and the prediction accuracy of the modified Fillippone method is 98.63%, which can accurately predict the pore pressure of the Maokou Formation.

**Table 4:** Error analysis for pore pressure results predicted by logging interpretation, Eaton method, and modified Fillippone method.

Horizon	Well name	Depth (m)	Result type	Pore pressure (MPa)	Relative error (%)
Maokou Formation	PS7	6038.76	Rock physics experiment	132.94	
			Logging explained	132.88	0.05
			Eaton Method (seismic prediction)	124.66	6.22
			Modified Fillippone method (seismic prediction)	132.21	0.55
	PY1	6019.98	Rock physics experiment	132.45	
			Logging explained	132.44	0.01
			Eaton Method (seismic prediction)	120.73	8.85
			Modified Fillippone method (seismic prediction)	130.70	1.32
	PS9	5746.39	Rock physics experiment	126.45	
			Logging explained	126.35	0.08
			Eaton Method (seismic prediction)	113.58	10.18
			Modified Fillippone method (seismic prediction)	123.61	2.25

**Figure 10:**
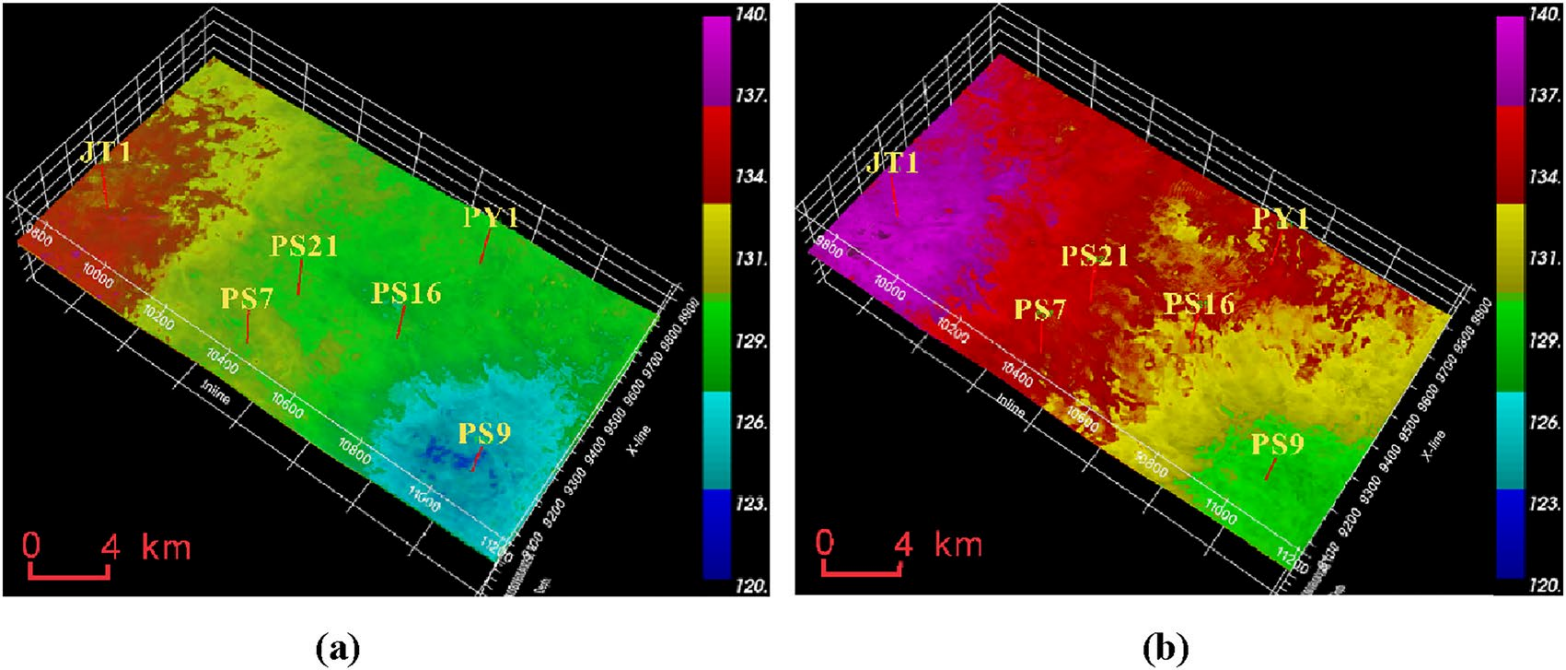
Formation pore pressure data volume of Maokou Formation calculated based on seismic data. (**a**) Pore pressure of Mao 2 Member-Mao 3 Member. (**b**) Pore pressure of Mao 1 Member.

From the predicted data of pore pressure in Fig. 10, it can be observed that the pore pressure in the Mao 2 Member-Mao 3 Member is between 120 and 140 MPa, with the highest values concentrated near the JT1 well at around 135 MPa and the lowest values in the PS9 well area at around 120 MPa. The pore pressure in the Mao 1 Member is between 130 and 145 MPa, significantly higher than the Mao 2 Member-Mao 3 Member. The JT1 well area has the highest pore pressure at around 145 MPa, while the PS9 well area has the lowest pore pressure at around 130 MPa.

**Figure 11:**
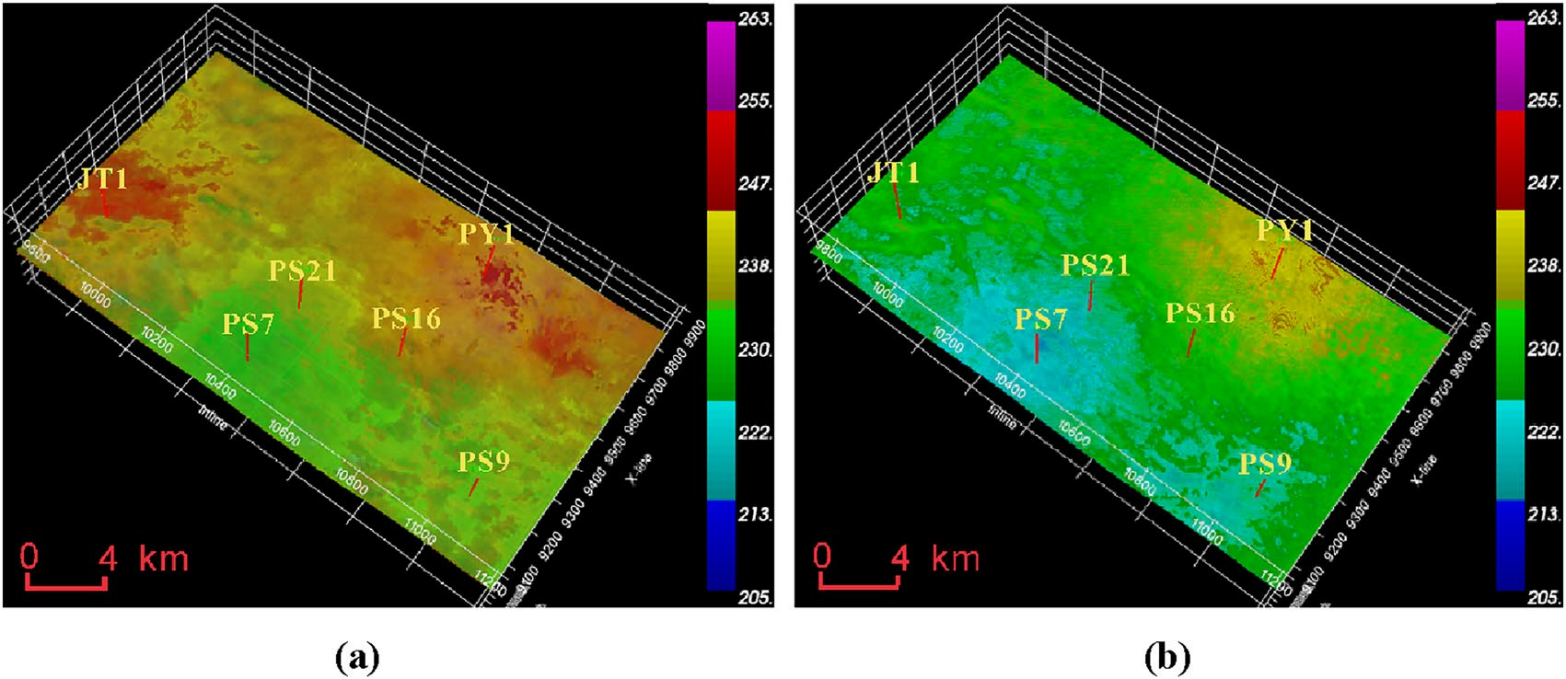
Formation fracture pressure data volume of Maokou Formation calculated based on seismic data. (**a**) Fracture pressure of Mao 2 Member-Mao 3 Member. (**b**) Fracture pressure of Mao 1 Member.

From Fig. 11, it can be seen that the fracture pressure of the Mao 2 Member-Mao 3 Member is between 220 and 250 MPa. The high-pressure zone is located near Well JT1 and PY1, around 250 MPa, while the PS7 well area has the lowest pressure, around 230 MPa. The fracture pressure of the Mao 1 Member is generally lower than that of the Mao 2 Member and Mao 3 Member, ranging from 210 to 240 MPa. The PY1 well area has the highest pressure, while the PS7 well area has the lowest pressure.

**Figure 12:**
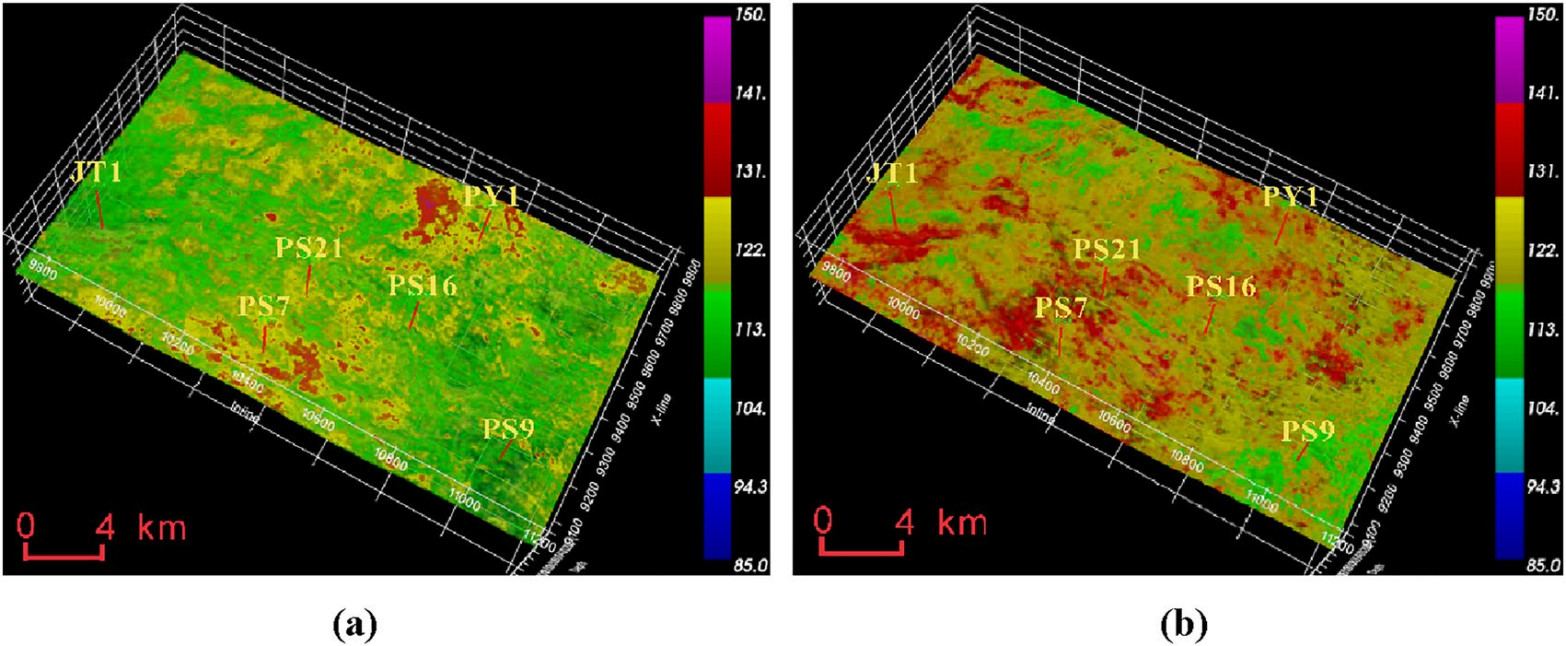
Formation collapse pressure data volume of Maokou Formation calculated based on seismic data. (**a**) Collapse pressure of Mao 2 Member-Mao 3 Member. (**b**) Collapse pressure of Mao 1 Member.

From Fig. 12, it can be seen that the collapse pressure of the Mao 2 Member-Mao 3 Member is between 95 and 125 MPa, with the highest value in the PY1 and PS7 well areas, around 125 MPa, and slightly lower in the PS9 well area, around 110 MPa; the collapse pressure of the Mao 1 Member is generally higher than that of the Mao 2 Member-Mao 3 Member, between 110 and 150 MPa, with the lowest value in the PS9 well area, around 115 MPa.

**Figure 13:**
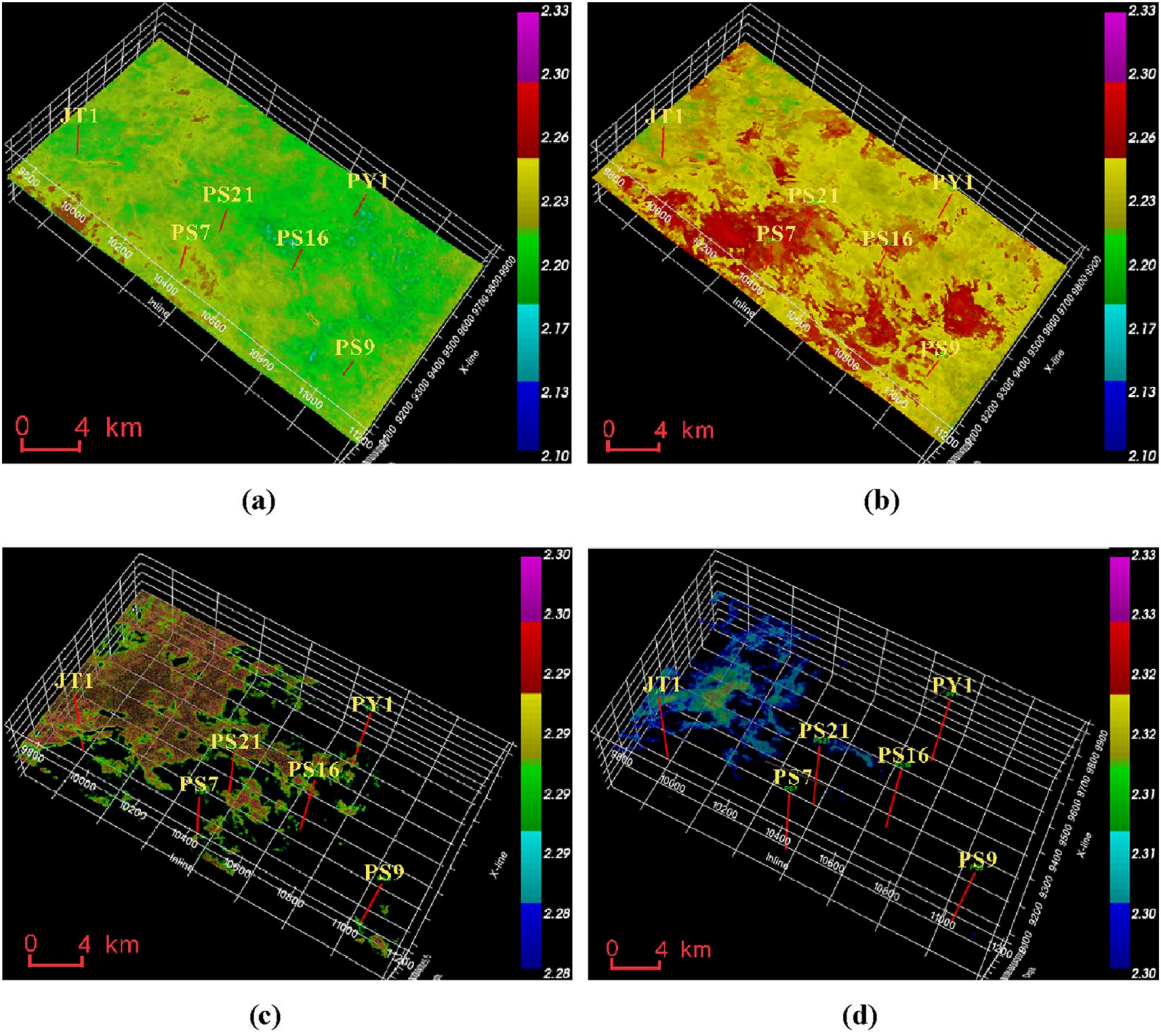
Maokou Formation formation pore pressure coefficient seismic predictor data display. (**a**) Pressure coefficient body of Mao 2 Member-Mao 3 Member. (**b**) Pressure coefficient body of Mao 1 Member. (**c**) The distribution of anomalous areas with a pressure coefficient greater than 2.28. (**d**) The distribution of anomalous areas with a pressure coefficient greater than 2.3.

The pressure coefficient of the Maokou Formation in Fig. 13 shows that the pressure coefficient of the Mao 2 Member-Mao 3 Member is between 2.1 and 2.26, which is slightly higher near well PS9, well PS 16 and well PS 21, and lower near well JT1. The pressure coefficient of the Mao 1 Member is between 2.16 and 2.3, which is slightly higher than that of the Mao 2 Member-Mao 3 Member, and the pressure coefficient near well PS7, PS16 and PS21 is larger (above 2.25), and the pressure coefficient near well JT1 is slightly lower. Transversely, the high-pressure strata with a pressure coefficient greater than 2.28 in the Maokou Formation are distributed in the area north of the JT well area, and the high-pressure strata with a pressure coefficient greater than 2.3 are distributed in a small part of the west of the work area.

From the above pressure prediction results, it can be seen that the Maokou Formation belongs to an abnormally high-pressure formation and requires a high drilling fluid density. According to the theory of mechanical stability of wellbore walls, in order to prevent the rock on the wellbore from peeling off and causing wellbore deformation, the liquid column pressure generated by drilling fluid must be higher than the maximum pore pressure and collapse pressure of the formation, and lower than the fracture stress of the formation. Therefore, the density range of drilling fluid can be determined based on the above prediction results.

## Coupling relationship between drilling events and pressure prediction

**Figure 14:**
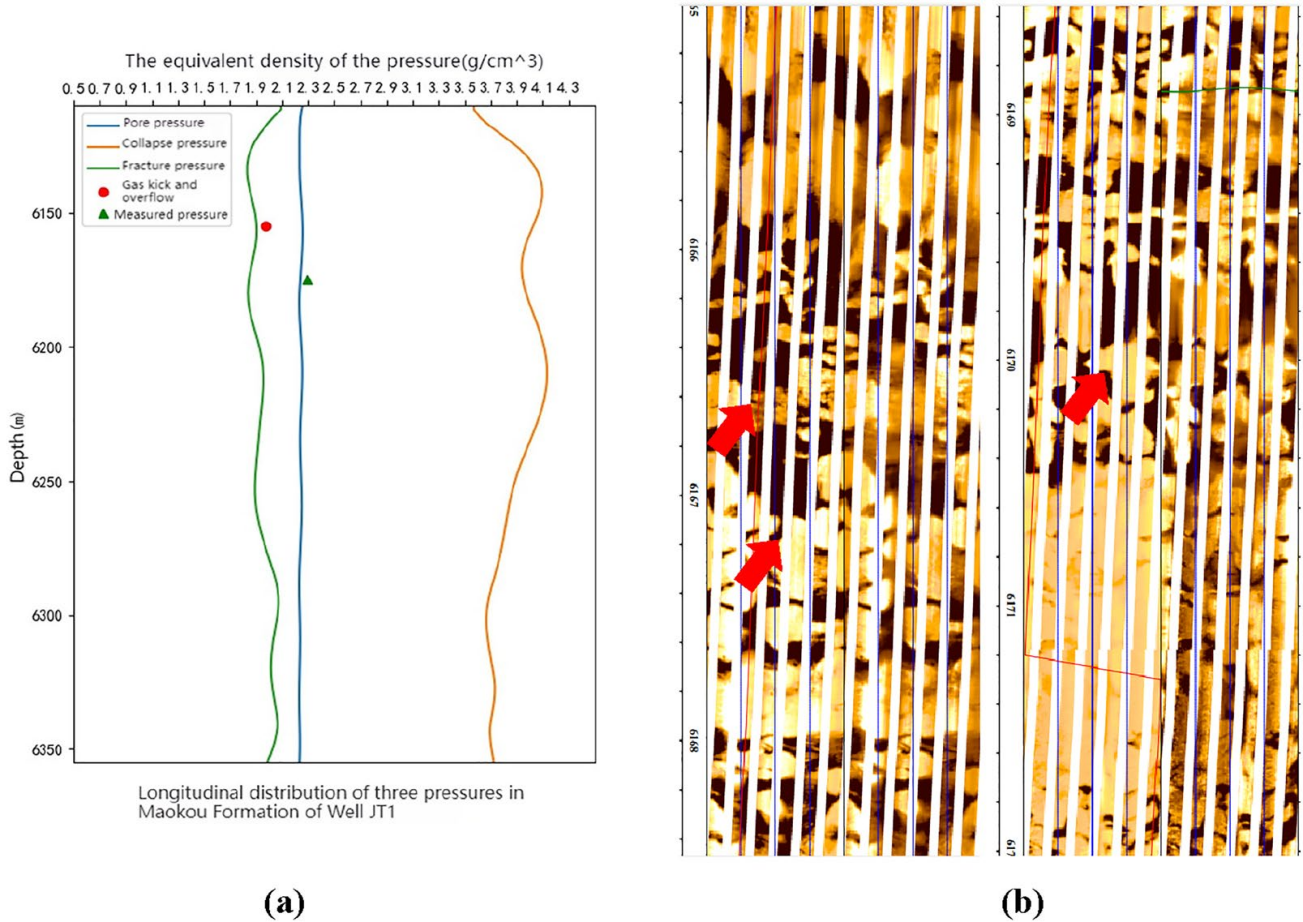
The coupling relationship between drilling events and pressure prediction in the Maokou Formation of Well JT1 and Well PY1. (**a**) Prediction results of formation three-pressures of the Maokou Formation in Well JT1. (**b**) PY1 well 6165–6172 m imaging log.

In order to further verify the feasibility and correctness of the calculation method in this article, a coupling relationship analysis was conducted between drilling events and predicted data volume. As shown in Fig. 14a, the drilling fluid density used in the Maokou Formation of well JT1 at 6158.98 m was 1.96 g/cm^3^. The equivalent density of pore pressure converted from the predicted results of the three pressures in this article is about 2.21 g/cm^3^, which is greater than the pressure of the drilling fluid. The fractures and caves in the Maokou Formation of the research area are more developed. When the pore pressure is greater than the drilling fluid pressure, gas overflows through the fractures and caves. After an overflow accident occurs, the density is increased to 2.35 g/cm^3^. After increasing the drilling fluid density and increasing the bottom hole pressure, the overflow stops. Then, the drilling fluid density gradually decreases to 2.30 g/cm^3^, and normal drilling is carried out.

For example, the Maokou Formation of Well PY1 experienced a gas kick event at 6165.50–6172.00 m. The drilling fluid density used in this section is 2.27 g/cm^3^. The equivalent density of the fracturing pressure predicted by the three-pressure conversion is 3.0 g/cm^3^, and the equivalent density of the formation pore pressure is 2.21 g/cm^3^. Combined with imaging logging (Fig. 14b), it can be observed that the leakage section has karst development (indicated by red arrows), forming natural leakage channels. The drilling fluid freely flows into the formation under the pressure difference, resulting in drilling fluid loss.

**Figure 15:**
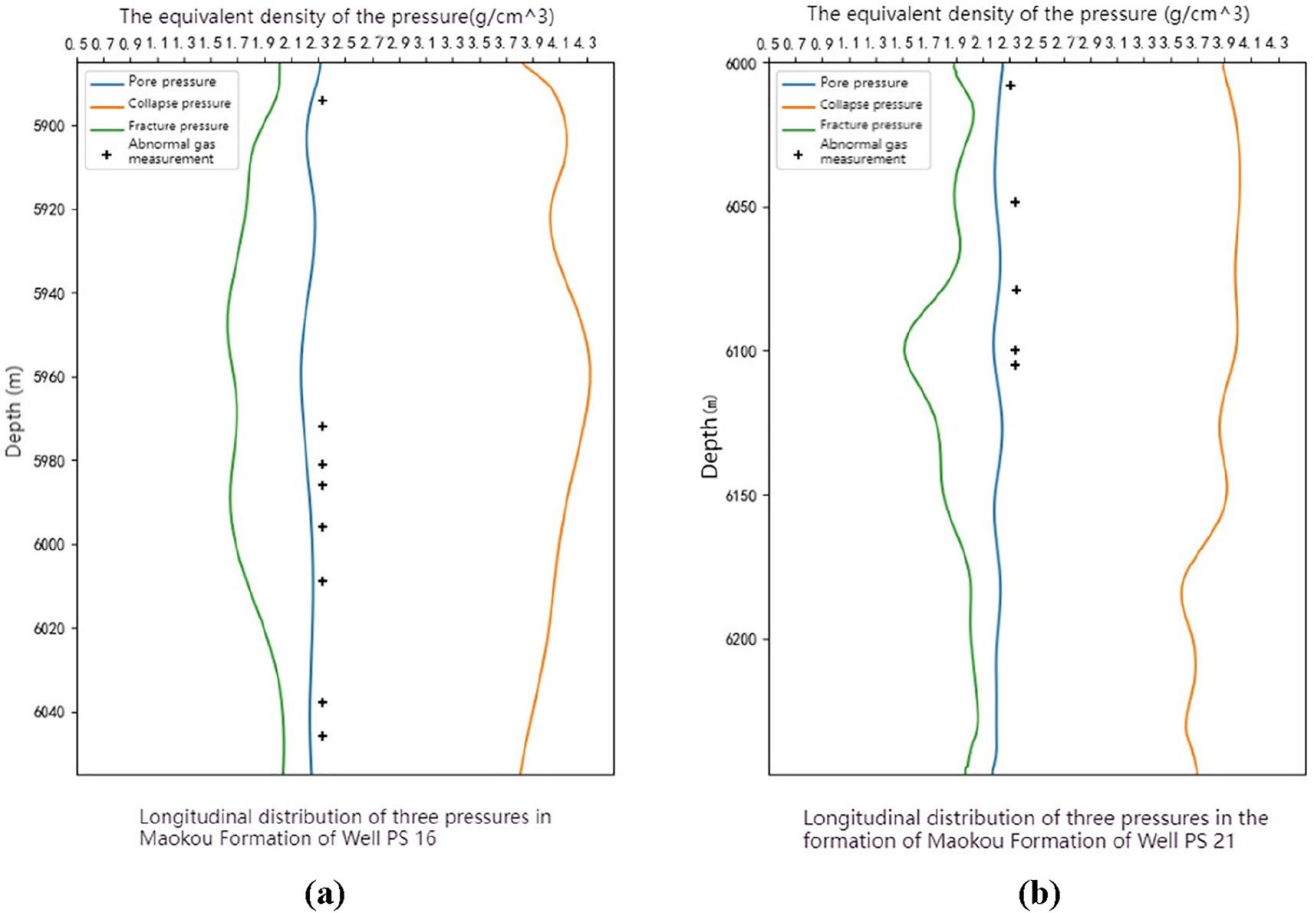
The coupling relationship between drilling events and pressure prediction in the Maokou Formation of well PS16 and well PS21. (**a**) Three-pressure prediction results from well PS16. (**b**) Three-pressure prediction results from well PS21.

The three pressure prediction results of the Maokou Formation in the well PS16 and well PS21 in the research area indicate that the pore pressure equivalent density ranges from 2.24 to 2.32 g/cm^3^. The on-site drilling fluid density was reasonably chosen higher than the values in this range, and there were no severe gas invasion or overflow events during the drilling operation. The drilling fluid pressure was close to the pore pressure in positions where the drilling fluid density was close to the predicted values, and some gas entered the drilling fluid, leading to several gas anomaly events. These phenomena further demonstrate the high degree of consistency between the predicted results in this study and the engineering reality, providing evidence for the correctness of the predictions (Fig. 15).

The high coupling between the three-pressure prediction results and the drilling events indicates that the three-pressure prediction results in this paper have high reliability. As can be seen from Figs. 14a, 15a,b, the column pressure generated by the density of the drilling fluid needs to be greater than the pore pressure and less than the rupture pressure. It can be seen from Fig. 14a that below the depth of 6215 m in Well JT1, the fracture pressure generally decreases with the increase of depth, resulting in a narrowing of the drilling fluid density window. Therefore, in the actual drilling process, the design of drilling fluid density needs to be more accurate.

## Conclusions

1. The Maokou Formation in the study area has a large number of dissolution holes, micro-fractures, cleavage planes and other weak structural features, and the dissolution pores are connected by micro-fractures, forming drilling fluid leakage channels and reservoir spaces, and the drilling fluid pressure is greater than the formation pore pressure or less than the fracture pressure, leading to drilling events such as well leakage and borehole collapse.
2. The Maokou Formation in the research area is characterized by abnormally high pressure, making it susceptible to gas invasion, well kicks, and even blowout accidents. To balance the high formation pressure, high-density drilling fluid is required.
3. The three-pressure prediction method of jointing well-seismic data can predict the formation longitudinal and transverse pressure anomaly zones in advance, which provides an important basis for the geological design of drilling and the disposal plan of drilling events, and can help the efficient implementation of drilling in complex areas or at deep and ultra-deep depths.

## Data Availability

The data used to support the findings of this study are available from the corresponding author upon request.
